# Identification of key symptoms impacting HRQOL in advanced renal cell carcinoma: nivolumab plus cabozantinib versus sunitinib in CheckMate 9ER

**DOI:** 10.1093/oncolo/oyag329

**Published:** 2026-08-14

**Authors:** Cristiane Bergerot, Shengsheng Yu, Min Yang, Bruno Martins, Mengtian Du, Jose Perez Torrealba, Tasha Hall, Navendu Samant, Robert J Motzer, David Cella

**Affiliations:** Oncoclínicas&Co—Medica Scientia Innovation Research (MEDSIR), Sao Paulo 01309-001, Brazil; Exelixis, Inc., Alameda, CA 94502, United States; Analysis Group, Inc., Boston, MA 02199, United States; Analysis Group, Inc., Boston, MA 02199, United States; Analysis Group, Inc., Boston, MA 02199, United States; Exelixis, Inc., Alameda, CA 94502, United States; Exelixis, Inc., Alameda, CA 94502, United States; Exelixis, Inc., Alameda, CA 94502, United States; Memorial Sloan Kettering Cancer Center, New York, NY 10065, United States; Feinberg School of Medicine, Northwestern University, Chicago, IL 60611, United States

**Keywords:** nivolumab, cabozantinib, advanced renal cell carcinoma, health-related quality of life, patient-reported outcomes

## Abstract

**Background:**

Nivolumab plus cabozantinib demonstrated significantly better clinical and patient-reported outcomes (PROs) compared with sunitinib as first-line therapy for advanced renal cell carcinoma (aRCC) in CheckMate 9ER. This post-hoc analysis identified key factors contributing to improvements in health-related quality of life (HRQOL) in these patients.

**Materials and Methods:**

Mediation analyses were conducted using first-year CheckMate 9ER data to delineate treatment effects as reflected by HRQOL improvements measured with the EuroQoL 5-Dimension 3-Level questionnaire (EQ-5D-3L) and 19-item Functional Assessment of Cancer Therapy-Kidney Symptom Index (FKSI-19) Functional Well-Being Subscale (FKSI-FWB). The incremental benefits of nivolumab plus cabozantinib over sunitinib were partitioned into mediated effects through symptom and emotional well-being improvements (FKSI-19 scales).

**Results:**

Nivolumab plus cabozantinib (*n *= 323) resulted in significantly greater improvement in EQ-5D utility versus sunitinib (*n *= 328; difference = 0.08 [95% confidence interval [CI], 0.05-0.12]) and numerically greater FKSI-FWB improvement (difference = 0.22 [95% CI, –0.19 to 0.63]), both primarily driven by greater reductions in fatigue, pain, shortness of breath, and cough, and improvements in emotional well-being (all *P *< 0.05). Reduced fatigue was the largest contributor to gains in both scales (24.0% and 21.1% of total mediated effect, respectively); improvement in emotional well-being accounted for 5.0% and 17.2% of the total mediated effect.

**Conclusion:**

These findings highlight underlying pathways contributing to HRQOL improvement in patients with aRCC receiving nivolumab plus cabozantinib. These data suggest that treatment with nivolumab plus cabozantinib is associated with an improvement in symptoms that can be quantified using PRO measures, supporting the utility of incorporating patient-centric outcomes into treatment decision making.

**ClinicalTrials.gov:**

NCT03141177

Implications for practice In the CheckMate 9ER trial, patients with advanced renal cell carcinoma treated with nivolumab plus cabozantinib had greater improvements in health-related quality of life (HRQOL) versus those receiving sunitinib. This was demonstrated using patient-reported outcome (PRO) measures. HRQOL improvements were driven by greater reduction in fatigue, pain, shortness of breath, and cough, and improvements in emotional well-being—areas that directly impact patients’ lives. Treatments that result in effective symptom management contribute to meaningful gains in quality of life, highlighting the importance of taking PRO measures into account when making treatment decisions.

## Introduction

Renal cell carcinoma (RCC) is the most common malignant kidney neoplasm^1^; it is estimated to account for 3% of all cancers worldwide, with over 431,000 new cases diagnosed annually.^2^ Over the last 3 decades, the widespread use of imaging has enabled earlier detection of RCC^3^^,^^4^; however, nearly a third of patients still present with advanced RCC (aRCC) and face a poor prognosis.^1^^,^^5^ Despite therapeutic advances, aRCC remains associated with considerable morbidity and mortality, with a 5-year survival rate estimated at just 5-12%.^5–7^

Patients with aRCC frequently experience a broad range of disease-related symptoms, including pain, fatigue, shortness of breath, cough, weight loss, and hematuria.^6^^,^^8–10^ Symptoms of aRCC are often present at diagnosis and may worsen with disease progression.^8^^,^^10^ Beyond their physical burden, symptoms of aRCC can substantially impair patients’ health-related quality of life (HRQOL) by limiting daily functioning, reducing the ability to work or engage in social activities, and contributing to emotional distress and worry about disease progression.^8^^,^^11^ As a result, the relationship between symptom burden and HRQOL has become an increasingly important consideration in the management of aRCC, alongside traditional efficacy outcomes such as progression-free survival (PFS), overall survival (OS), and objective response rate (ORR).

In recent years, the introduction of immune checkpoint inhibitors in combination with vascular endothelial growth factor-targeted therapies has transformed the first-line treatment landscape for aRCC. Notably, the phase 3 CheckMate 9ER trial (NCT03141177) in patients with previously untreated aRCC demonstrated that nivolumab plus cabozantinib significantly improved clinical efficacy outcomes compared with sunitinib, including PFS (median, 16.6 vs 8.3 months; hazard ratio [HR], 0.51), OS (85.7% vs 75.6% at 12 months; HR, 0.60), and ORR (55.7% vs 27.1%).^12^ These benefits were observed across International Metastatic Renal Cell Carcinoma Database Consortium (IMDC) risk groups.

In addition to conventional efficacy outcomes, nivolumab plus cabozantinib has shown favorable patient-reported outcomes (PROs) in post-hoc analyses of CheckMate 9ER, including improvements in HRQOL over time relative to sunitinib.^13^^,^^14^ Prior exploratory PRO analyses found that, compared with sunitinib, patients with intermediate or poor IMDC risk score treated with nivolumab plus cabozantinib had improved or stable HRQOL.^15^ Although these benefits have been reported elsewhere, the mechanisms underlying improvements in HRQOL were not well understood in the context of RCC management. In particular, while patients may experience disease-specific symptom alleviation resulting from the effectiveness of a treatment, it is unclear to what extent such improvements may in turn explain the benefits reflected in the PRO data indicating improved HRQOL.

Mediation analysis is a type of regression modeling. It has been used to disentangle and quantify the extent to which benefits of a treatment on an outcome such as HRQOL can be explained by intermediate mediator variables (eg, symptoms).^16^^,^^17^ This post-hoc analysis of the CheckMate 9ER trial aimed to use mediation analysis to identify and analyze the key factors that contributed to improved HRQOL with nivolumab plus cabozantinib versus sunitinib.

## Materials and methods

### Study design and data source

Details on the design and statistical analysis of the phase 3, randomized, open-label CheckMate 9ER trial comparing nivolumab plus cabozantinib with sunitinib monotherapy in patients with aRCC (NCT03141177) have previously been reported.^12^^,^^13^ The trial was approved by the institutional review board or an ethics committee at each site and was conducted in accordance with Good Clinical Practice guidelines defined by the International Council for Harmonization. Enrolled patients provided written informed consent according to the principles of the Declaration of Helsinki.

**Figure 1.**
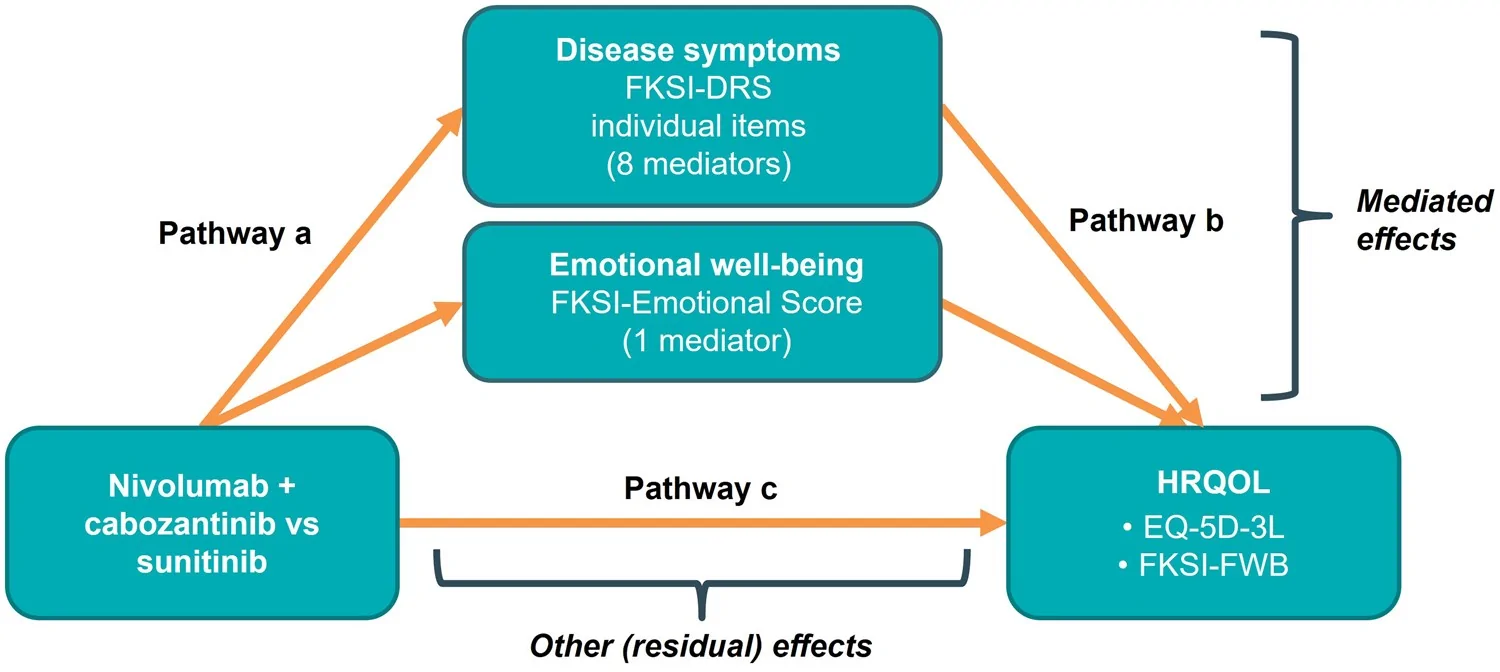
Schema for the treatment effect of nivolumab plus cabozantinib on HRQOL measured by the EQ-5D-3L questionnaire and FKSI-19 scales. EQ-5D-3L, EuroQol 5-Dimension 3-Level questionnaire; FKSI-19, 19-item Functional Assessment of Cancer Therapy-Kidney Symptom Index; FKSI-DRS, Disease Related Symptoms of the FKSI-19; FKSI-FWB, Functional Well-Being Subscale of the FKSI-19; HRQOL, health-related quality of life.

In this post-hoc analysis of individual patient-level data from the CheckMate 9ER trial, mediation analysis was used to parse out and estimate the effects of disease-specific symptoms and associated emotional well-being on the observed improvements in patient-reported HRQOL with nivolumab plus cabozantinib versus sunitinib. The analytic framework is illustrated in Figure 1. Conceptually, the mediation analysis framework intends to explain that when it comes to less tangible outcomes such as HRQOL, treatment effects are most likely manifested through indirect impacts of the more directly measurable outcomes (eg, symptoms acting as mediators). Specifically, Figure 1 shows that treatments have a direct effect on the mediators (Pathway a), and the mediators, in turn, affect HRQOL (Pathway b). The indirect (mediated) effect is then estimated as the product of both Pathways (a *×* b), while the remaining/residual effect represents the treatment effect not explained by the mediators (Pathway c).

### Population

Adult patients enrolled in CheckMate 9ER had measurable disease according to Response Evaluation Criteria in Solid Tumors version 1.1, either metastatic RCC or aRCC not amenable to curative surgery or radiation therapy, any IMDC risk score, and a Karnofsky performance-status score of ≥70. Patients were randomized 1:1 to receive nivolumab 240 mg intravenously every 2 weeks plus cabozantinib 40 mg orally once daily, or sunitinib 50 mg orally once daily for 4 weeks, followed by 2 weeks off (6-week cycle), for a maximum of 2 years. The primary endpoint was PFS, and the secondary endpoints were OS, ORR, and safety.

### PROs and assessment schedule

This analysis used the same dataset as prior PRO analyses conducted for CheckMate 9ER, with a database lock date of September 10, 2020 (median follow-up, 23.5 months [interquartile range, 21.0-26.5]). PRO data were collected more frequently in the nivolumab plus cabozantinib group (every 2 weeks) than in the sunitinib group (every 6 weeks). To enable valid comparisons at common time points, this analysis used PRO data collected at 6-week intervals in both treatment arms.

### HRQOL measures

HRQOL measures included the EuroQoL 5-Dimension 3-Level questionnaire (EQ-5D-3L)^13^ and the 19-item Functional Assessment of Cancer Therapy-Kidney Symptom Index (FKSI-19).^18^

#### EQ-5D-3L

The EQ-5D-3L is a PRO instrument for describing general health status through 5 questions/dimensions (mobility, self-care, usual activities, pain or discomfort, and anxiety or depression).^19^ Each dimension is evaluated using 3 levels ranging from no problems to severe/extreme problems. A utility index score (EQ-5D) is generated following an established algorithm to reflect a person’s general health state, where a value of 1 indicates full health, 0 indicates death, and less than 0 indicates a health state worse than death.^20^

#### FKSI-19

The FKSI-19 is a PRO instrument designed to measure disease-specific symptoms and HRQOL in patients with kidney cancer, using 5-point Likert-type response options.^18^ It contains 19 items with a 7-day recall period, covering key physical symptoms (eg, fatigue, pain, appetite/weight loss), emotional concerns, treatment-related side-effects, and overall functional well-being. Scores are summed, with higher scores indicating better symptom status and HRQOL. The Functional Well-Being Subscale of the FKSI-19 (FKSI-FWB) includes 3 of the 19 items, capturing the patient’s ability to work, ability to enjoy life, and contentment with the quality of their life. The summed subscale score ranges from 0 to 12, with a higher score indicating better functional well-being.

### Candidate mediators: disease symptoms and emotional well-being

Potential mediators of the treatment effect on HRQOL were selected *a priori* based on clinical relevance and availability in the PRO dataset.

#### Disease-related symptoms

Disease-related symptoms were assessed using individual items of FKSI-19-Disease Related Symptoms (FKSI-DRS). FKSI-DRS includes 9 items measuring symptoms of lack of energy, fatigue, pain, shortness of breath, bone pain, cough, fever, weight loss, and blood in urine. Each item is rated on a 0-4 scale, with higher values indicating more severe symptoms. Pairwise correlations among the symptoms were examined using baseline data through a Spearman correlation matrix (Table S1). Because lack of energy was highly correlated with fatigue, it was excluded to avoid redundancy. Accordingly, 8 symptom items were evaluated as candidate mediators.

#### Emotional well-being

Emotional well-being was evaluated with the FKSI-19-Emotional Subscale, which includes one item (emotion) that asks whether a person is worried about their condition getting worse. The score ranges from 0 to 4, with a higher score indicating better emotional well-being.

### Statistical analysis

Multilevel linear mixed-effect models were developed to partition the incremental effect of nivolumab plus cabozantinib over sunitinib on HRQOL into mediated effects from improvements in individual disease symptoms and emotional well-being over time. This approach allowed assessment of the entire trajectory and time trend of the outcomes and the longitudinal mediation effects. All models assumed that mediators were causally unrelated to each other.

For each outcome of interest, 2 regression models were developed to capture Pathways a and b separately: (1) a model assessing the association between treatment and the mediator (Pathway a), and (2) a model assessing the association between the mediator and HRQOL, adjusting for treatment (Pathway b) (Figure 1). The mediated effects were calculated as the product of the corresponding coefficients to capture the Pathways a *×* b, with 95% confidence intervals (CIs) generated through quasi-Bayesian Monte Carlo simulations to assess statistical significance.

All models were adjusted for baseline covariates including age, sex, race (White vs non-White), geographic region (United States, Europe, and rest of the world), baseline IMDC prognostic risk score (favorable [0], intermediate [1-2], poor [3-6]), and tumor programmed death-ligand 1 (PD-L1) expression (≥1%, <1%, indeterminate).

The analysis used as-observed, unimputed, individual patient-level data from baseline to week 49, the last common time point between treatment groups in the first year follow-up, selected to balance sufficient follow-up time to detect HRQOL effects while minimizing potential biases from attrition and loss of statistical power. Previous analyses of PROs in CheckMate 9ER showed that results were similar when using different assumptions on missing patterns.^13^ Therefore, no missing data imputation was used. Although long-term follow-up is available for HRQOL, use of the week 49 data was intended to reduce the potential survival bias associated with the later follow-up period.

The contribution of each individual mediator on the differences observed between treatment arms on HRQOL was then estimated as the proportion of the mediated effect of a selected contributor out of the sum of the collective mediated effects of all identified contributors. Analyses were conducted in R (version 4.4; The R Foundation, Vienna, Austria) using the “mediation” package.

## Results

### Patient population and baseline characteristics

A total of 651 patients were included in the analysis, with 323 randomized to nivolumab plus cabozantinib and 328 randomized to sunitinib. Baseline patient characteristics were balanced between the 2 treatment arms (Table 1). The mean age was 61.4 years (standard deviation [SD], 10.2) in the nivolumab plus cabozantinib arm and 60.4 years (SD, 10.6) in the sunitinib arm. Males comprised 77.1% and 70.7% of patients in the respective arms. IMDC risk categories were similar, with favorable-risk disease in 22.9% and 22.0%, intermediate-risk in 58.2% and 57.3%, and poor-risk in 18.9% and 20.7% of patients in the nivolumab plus cabozantinib and sunitinib arms, respectively. Tumor PD-L1 expression ≥1% was present in 25.7% and 25.3% of patients, respectively.

**Table 1.** Baseline characteristics of patients in CheckMate 9ER.

	Nivolumab plus cabozantinib	Sunitinib
	***n *= 323**	***n *= 328**
**Age, mean (SD) years**	61.4 (10.2)	60.4 (10.6)
**Sex, *n* (%)**		
**Male**	249 (77.1)	232 (70.7)
**Female**	74 (22.9)	96 (29.3)
**Geographic region, *n* (%)**		
**United States or Europe**	158 (48.9)	161 (49.1)
**Rest of the world**	165 (51.1)	167 (50.9)
**IMDC prognostic risk score, *n* (%)**		
**Favorable: 0**	74 (22.9)	72 (22.0)
**Intermediate: 1-2**	188 (58.2)	188 (57.3)
**Poor: 3-6**	61 (18.9)	68 (20.7)
**Tumor PD-L1 expression, *n* (%)**		
**≥1%**	83 (25.7)	83 (25.3)
**<1% or indeterminate**	240 (74.3)	245 (74.7)
**EQ-5D-3L, mean (SD)**		
**Utility index**	0.78 (0.25)	0.73 (0.29)
**FKSI-19, mean (SD)**		
**Total score (range, 0-76)**	58.74 (10.57)	58.39 (9.92)
**Functional Well-Being (range, 0-12)**	7.17 (3.49)	7.08 (3.48)
**Disease Related Symptoms items (range, 0-4)**		
**“I Am Bothered by Fevers”**	0.18 (0.59)	0.17 (0.61)
**“I Am Losing Weight”**	0.65 (1.05)	0.63 (1.01)
**“I Feel Fatigued”**	0.96 (1.07)	1.08 (1.04)
** “I Have Been Coughing”**	0.62 (1.00)	0.66 (0.96)
** “I Have Been Short of Breath”**	0.55 (0.91)	0.53 (0.91)
** “I Have Bone Pain”**	0.62 (0.97)	0.60 (1.01)
**“I Have Had Blood in My Urine”**	0.10 (0.51)	0.05 (0.28)
**“I Have Pain”**	0.96 (1.06)	1.01 (1.08)
**“I Have a Lack of Energy”**	1.11 (1.09)	1.20 (1.11)

Abbreviations: EQ-5D-3L, EuroQoL 5-Dimension 3-Level questionnaire; FKSI-19, 19-item Functional Assessment of Cancer Therapy-Kidney Symptom Index; IMDC, International Metastatic Renal Cell Carcinoma Database Consortium; PD-L1, programmed death-ligand 1.

Baseline HRQOL measures were also comparable between arms (Table 1). The mean EQ-5D utility scores were 0.78 (SD, 0.25) in the nivolumab plus cabozantinib group and 0.73 (SD, 0.29) in the sunitinib group; the FKSI-FWB scores were 7.17 (SD, 3.49) and 7.08 (SD, 3.48), respectively. Baseline disease-related symptom scores were generally low to moderate and similar across treatment groups.

### Longitudinal HRQOL trajectories

**Figure 2.**
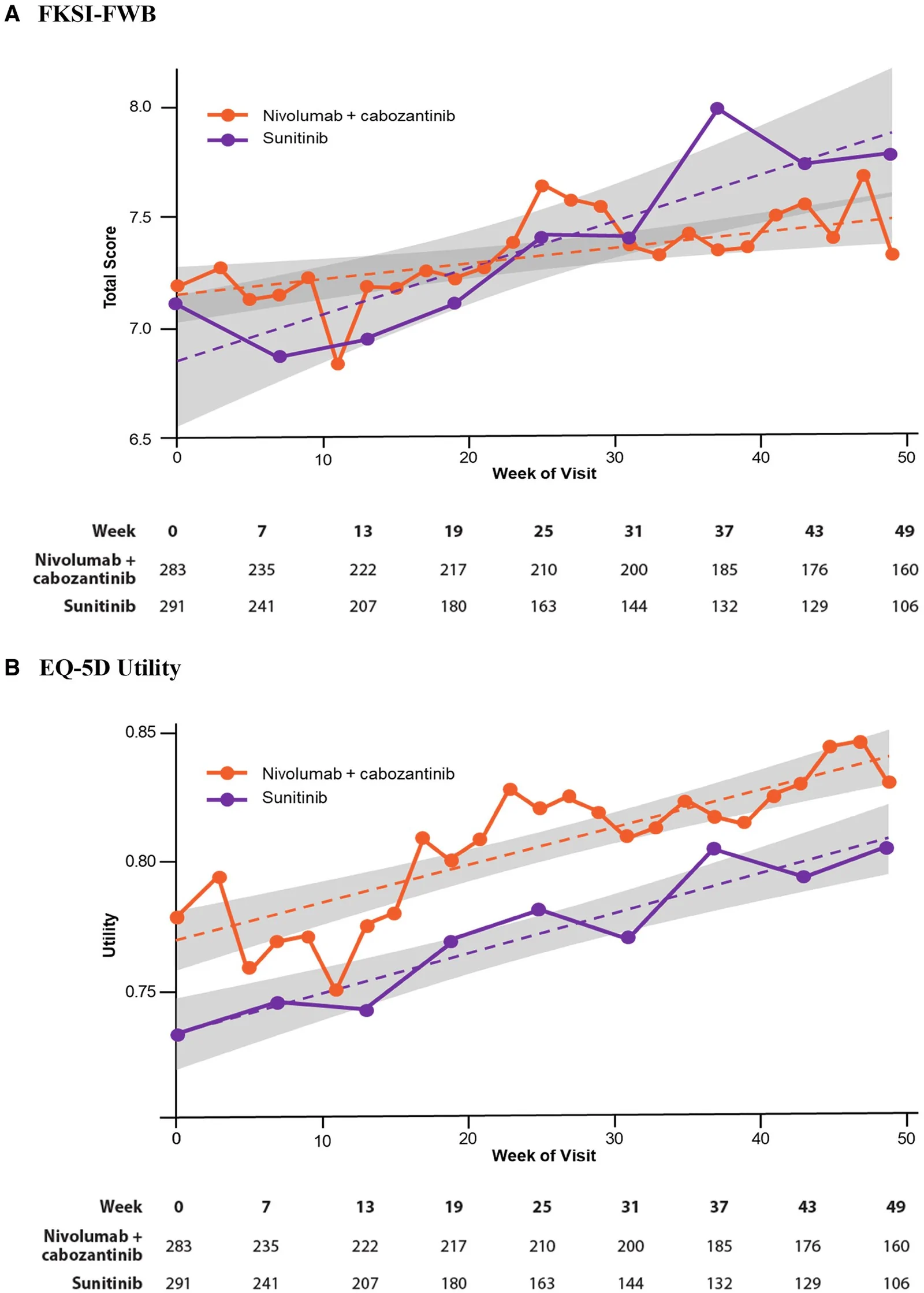
Time trajectory of HRQOL measures. Notes: All time points as collected in CheckMate 9ER through week 49 are shown. Solid dots indicate unimputed mean scores at each week of visit. Dashed lines are linear regression lines showing an unadjusted relationship between scores and week of visit. Shaded areas show 95% confidence intervals for predicted scores based on a linear regression model. EQ-5D-3L, EuroQol 5-Dimension 3-Level questionnaire; FKSI-FWB, Functional Well-Being Subscale of the 19-item Functional Assessment of Cancer Therapy-Kidney Symptom Index.

From baseline through week 49, both treatment arms showed improvements in HRQOL, with nivolumab plus cabozantinib associated with higher mean HRQOL scores versus sunitinib (Figure 2A and B). For EQ-5D utility scores, higher mean values were consistently observed in the nivolumab plus cabozantinib arm at all treatment assessments. For FKSI-FWB, mean scores in the nivolumab plus cabozantinib arm were numerically higher across early visits, up to week 30.

More PRO data were available for analysis in the nivolumab plus cabozantinib group, with 160 of 283 (56.5%) patients reporting on FKSI-19 and EQ-5D-3L by week 49, compared with 106 of 291 (36.4%) patients in the sunitinib group (Figure 2A and B). As previously reported, the reduced availability of PRO data over the course of the trial was due to treatment discontinuation, which was higher in the sunitinib group.^13^

### Changes in disease symptoms and emotional well-being

The regression models capturing Pathway a in Figure 1 are summarized in Table 2. Each row represents one model with the individual mediator as the outcome, and the coefficient of the treatment group (nivolumab plus cabozantinib vs sunitinib) as an explanatory variable is reported.

**Table 2.** Reduction in symptoms and improvement in emotional well-being in patients receiving nivolumab plus cabozantinib versus sunitinib over 49 weeks.

	**Estimated difference-in-differences between nivolumab plus cabozantinib vs sunitinib** ^a^ ^,^ ^b^
**Disease symptoms**	
**Fatigue**	−0.25*
**Pain**	−0.19*
**Cough**	−0.16*
**Shortness of breath**	−0.15*
**Bone pain**	−0.11
**Weight loss**	−0.08
**Fever**	−0.02
**Blood in urine**	−0.02
**Emotional well-being**	
**Emotion**	0.27*

a Negative values in disease symptoms are associated with symptom reduction in the nivolumab plus cabozantinib arm compared with sunitinib. Positive values in emotion are associated with an improvement in emotional well-being in the nivolumab plus cabozantinib arm, compared with sunitinib.

b Models include the following covariates: age (years), sex (male vs female), race (White vs non-White), geographic region (United States, Europe, and rest of the world), baseline IMDC prognostic risk score (favorable [0], intermediate [1-2], poor [3-6]), and tumor PD-L1 expression (≥1%, <1%, indeterminate).

* Statistically significant (*P *< 0.05).

Abbreviations: IMDC, International Metastatic Renal Cell Carcinoma Database Consortium; PD-L1, programmed death-ligand 1.

Over 49 weeks, nivolumab plus cabozantinib was associated with statistically significant reductions in multiple disease-specific symptoms versus sunitinib (Table 2). The largest improvement was observed for fatigue, followed by pain, cough, and shortness of breath (all *P *< 0.05). Improvements in bone pain and weight loss numerically favored nivolumab plus cabozantinib but were not statistically significant; changes in fever and blood in urine were minimally different between the treatment arms. Furthermore, emotional well-being improved significantly with nivolumab plus cabozantinib compared with sunitinib (*P *< 0.05).

These identified differences in patient-reported disease symptoms and emotional well-being between nivolumab plus cabozantinib versus sunitinib supported their strength in Pathway a and allowed further assessment of their intermediate contribution to clinical effects through Pathway b. Subsequently, mediation analyses were conducted to quantify the extent to which improvements in these individual disease symptoms and emotional well-being accounted for the observed treatment-associated differences in HRQOL.

### Mediated treatment effects on HRQOL

Mediation analyses demonstrated that reductions in disease symptoms and improvements in emotional well-being accounted for a substantial proportion of the observed HRQOL benefit with nivolumab plus cabozantinib compared with sunitinib. Table 3 summarizes the results of the mediated effects captured through Pathways a × b in Figure 1.

**Table 3.** Treatment effects of nivolumab plus cabozantinib versus sunitinib in HRQOL.

	**Estimated effects from the mediation models (95% CI)** ^a^	
	EQ-5D utility	FKSI-FWB
**Total effect of nivolumab plus cabozantinib vs sunitinib**	0.08 (0.05 to 0.12)*	0.22 (-0.19 to 0.63)
**Mediated effect by:**		
**Disease symptoms**		
**Fatigue**	0.02 (0.01 to 0.03)*	0.05 (0.02 to 0.09)*
**Pain**	0.01 (0.00 to 0.02)*	0.04 (0.01 to 0.08)*
**Cough**	0.01 (0.00 to 0.02)*	0.04 (0.01 to 0.07)*
**Shortness of breath**	0.01 (0.00 to 0.02)*	0.03 (0.01 to 0.06)*
**Bone pain**	0.01 (-0.00 to 0.02)	0.02 (-0.00 to 0.05)
**Weight loss**	0.01 (-0.00 to 0.01)	0.02 (-0.01 to 0.04)
**Fever**	0.00 (-0.00 to 0.01)	0.01 (-0.01 to 0.02)
**Blood in urine**	0.00 (-0.00 to 0.00)	0.00 (-0.00 to 0.01)
**Emotional well-being**		
**Emotion**	0.00 (0.00 to 0.01)*	0.04 (0.01 to 0.08)*
**Residual effect**	0.01 (-0.01 to 0.03)	−0.03 (-0.42 to 0.37)

a Models include the following covariates: age (years), sex (male vs female), race (White vs non-White), geographic region (United States, Europe, and rest of the world), baseline IMDC prognostic risk score (favorable [0], intermediate [1-2], poor [3-6]), and tumor PD-L1 expression (≥1%, <1%, indeterminate).

* Statistically significant (*P *< 0.05).

Abbreviations: CI, confidence interval; EQ-5D-3L, EuroQol 5-Dimension 3-Level questionnaire; FKSI-FWB, Functional Well-Being Subscale of the 19-item Functional Assessment of Cancer Therapy-Kidney Symptom Index; HRQOL, health-related quality of life; IMDC, International Metastatic Renal Cell Carcinoma Database Consortium; PD-L1, programmed death-ligand 1.

#### EQ-5D utility

**Figure 3.**
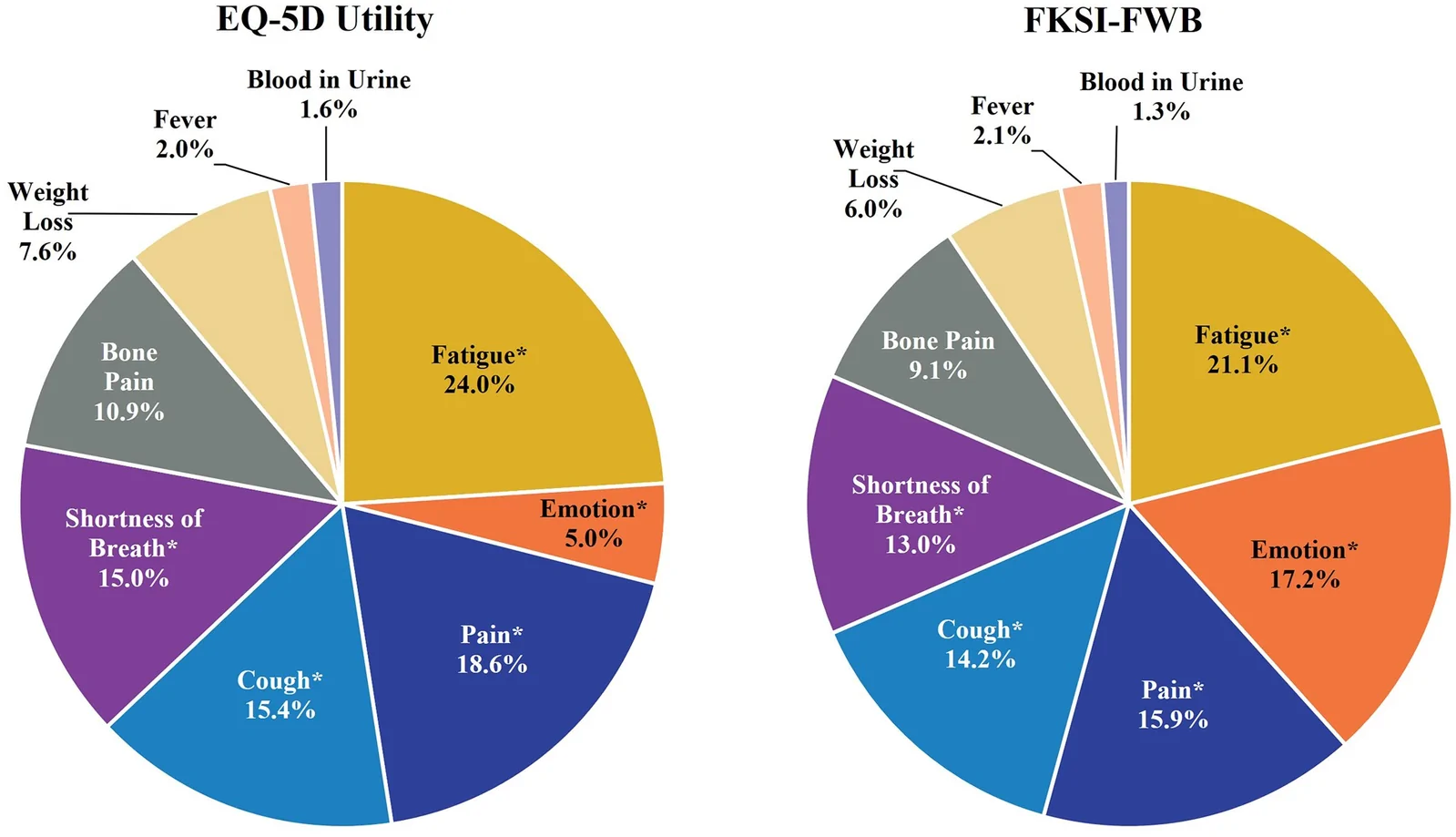
Distribution of the improvement in HRQOL mediated by improvements in disease symptoms and emotional well-being. Notes: Percentage of effect explained by each mediator was calculated as each individual mediation effect divided by the sum of the overall mediated effects. *Statistically significant values for the mediator (*P *< 0.05). EQ-5D, EuroQol 5-Dimension; FKSI-FWB, Functional Well-Being Subscale of the 19-item Functional Assessment of Cancer Therapy-Kidney Symptom Index.

The total difference-in-differences treatment effect of nivolumab plus cabozantinib versus sunitinib on EQ-5D utility was 0.08 (95% CI, 0.05-0.12) (Table 3). The incremental effect on EQ-5D utility was driven primarily by significant mediated effects from fatigue (24.0% of total mediated effect), pain (18.6%), cough (15.4%), and shortness of breath (15.0%; all *P *< 0.05) (Figure 3). Emotional well-being contributed a smaller but statistically significant mediated effect, accounting for 5.0% of the incremental improvements. Disease symptoms of bone pain, weight loss, fever, and blood in urine were associated with minimal, non-significant, incremental HRQOL benefits in the nivolumab plus cabozantinib group, accounting for 22.1% of the total mediated effect on EQ-5D utility.

#### FKSI-FWB

The total difference-in-differences treatment effect of nivolumab plus cabozantinib versus sunitinib on FKSI-FWB was 0.22 (95% CI, -0.19 to 0.63), which favored nivolumab plus cabozantinib but was not statistically significant (Table 3). Significant mediated effects were observed for fatigue (21.1% of total mediated effect), pain (15.9%), cough (14.2%), and shortness of breath (13.0%; all *P *< 0.05) (Figure 3). Improvement in emotional well-being in the nivolumab plus cabozantinib versus sunitinib group represented 17.2% of the total mediated effect (*P *< 0.05). Symptoms of bone pain, weight loss, fever, and blood in urine were associated with minimal, non-statistically significant, incremental benefits in the nivolumab plus cabozantinib group, accounting for 18.5% of the mediated effects on FKSI-FWB.

## Discussion

While the focus of cancer treatments remains on efficacy (eg, PFS and OS), HRQOL has become an important outcome in clinical trials. With advances in cancer therapies, maintaining and potentially improving patients’ HRQOL has gained increasing importance. In fact, 2 recent survey studies reported that patients with metastatic RCC ranked HRQOL as the third most important factor—after decreases in tumor size and achieving stable disease—for both treatment selection and defining treatment success.^21^^,^^22^ Understanding these relationships is clinically relevant for prescribers, as treatment selection in aRCC increasingly requires balancing efficacy, toxicity, and patient-centered outcomes. Insight into how specific symptom improvements translate into better HRQOL may help clinicians align therapeutic choices with patient priorities, set realistic expectations for treatment benefit, and optimize supportive-care strategies.

In this post-hoc analysis of CheckMate 9ER, patients treated with nivolumab plus cabozantinib had meaningful incremental improvements in HRQOL compared with those receiving sunitinib, and these benefits were largely explained by improvements in disease-specific symptoms and emotional well-being. Over the first year of the study period, patients receiving nivolumab plus cabozantinib versus sunitinib experienced greater reductions in key symptoms (fatigue, pain, cough, and shortness of breath) as well as enhanced emotional well-being. Mediation analyses demonstrated that these symptom improvements accounted for a substantial proportion of the observed HRQOL benefit, particularly for EQ-5D utility, for which the total incremental treatment effect was statistically significant. Reduction in fatigue emerged as the largest contributor to HRQOL improvement across measures, followed by pain, respiratory symptoms, and emotional well-being; in contrast, the residual effects of treatment on HRQOL were minimal and not statistically significant. The pathways identified were consistent across both the disease-specific (FKSI-FWB) and general (EQ-5D-3L) HRQOL measures. In addition, the ranking of the individual contributions of each disease symptom on the total mediated effect was also consistent across all HRQOL measures. Together, these findings suggest that the HRQOL advantages observed with nivolumab plus cabozantinib were driven primarily by improvements in symptom burden and emotional status rather than unexplained treatment effects.

Patients with aRCC face a large burden from their medical condition and experience a multitude of symptoms during the course of the disease that can lead to HRQOL deterioration.^6^^,^^8–11^ Nevertheless, the impact of aRCC and its treatments on HRQOL has historically been studied only superficially, without an in-depth evaluation of the driving factors behind changes in HRQOL. Understanding the underlying mechanisms through which patients’ HRQOL improves provides a meaningful interpretation of such PRO measures for practitioners, and this may in turn aid in the clinical management of their patients. For example, fatigue and pain are among the most commonly reported and impactful symptoms of aRCC, consistently associated with diminished functional well-being and overall HRQOL.^8^^,^^23–25^ Respiratory symptoms such as cough and shortness of breath may further compromise physical functioning, while systemic symptoms including weight loss and fever can reflect underlying disease activity.^26^ Emotional well-being, including anxiety or worry related to disease progression, represents an additional dimension of patient experience that is closely intertwined with physical symptoms and overall HRQOL. Collectively, these factors highlight the complex and multidimensional nature of symptom burden in aRCC and its central role in shaping PROs.

The items in the FKSI-19 identified in this study as contributing factors to the incremental benefits in HRQOL are consistent with previous research that identified fatigue/lack of energy, emotional well-being, and pain as the most relevant questions from the FKSI-19 for this population (with blood in urine and fever the least relevant).^27^ After the symptom of fatigue, the current study identified mental well-being as the second largest underlying mechanism causing changes in FKSI-FWB. Previous research using clinical trial data has shown that emotional distress may be a marker associated with clinical responses in patients with melanoma,^28–30^ and the current analysis further highlights the value of considering emotional well-being in the management of patients with aRCC.

PRO instruments such as the EQ-5D-3L measure general health status, which may encompass a broad range of patient experiences. The changes in its nominal scores often are not well understood, leading to difficulty in interpreting such findings from a clinical trial. This analysis demonstrates the clear connection between symptoms and their impact on such scores. To complement the EQ-5D-3L being a general HRQOL measure, our analysis also included the disease-specific FWB domain from the FKSI-19, which informs patient functional well-being specifically in the context of kidney cancer. A systematic literature review on psychological distress confirmed a high prevalence of depressive and anxiety symptoms (∼70% or higher) in patients with RCC; moreover, depression has been shown to have an impact on OS in the metastatic RCC population.^31^ Notably, in an earlier post-hoc analysis of PRO data, patients treated with nivolumab plus cabozantinib had significantly delayed time to deterioration in FWB compared with sunitinib over 2 years.^13^ In the current analysis, trial data were limited to week 49. Although the improvement in FWB only numerically favored nivolumab plus cabozantinib, FWB was considered in this context to inform the role of symptom changes on patients’ functional well-being.

Collectively, these analyses provide an interpretation for changes in PRO measures that are directly related to specific symptom alleviation and improvements in emotional well-being, all of which are directly relevant to the patient experience. This analysis combines these 2 concepts in a single framework, providing a robust and holistic depiction of treatment effects on HRQOL.

### Limitations

Although guided by a methodological framework, these analyses were exploratory in nature, and future studies are needed to confirm the findings. Multiple mediators were evaluated simultaneously under the assumption that mediators are independent. While symptoms that were highly correlated were excluded from being mediators, individual mediated effects may still not be uniquely identifiable, which could result in over‑attribution of observed HRQOL improvements to specific symptoms. Acknowledging the challenges of isolating the combined, interacting effects of multiple, potentially overlapping symptoms on HRQOL, baseline pairwise correlations were conducted across symptoms from the FKSI-DRS. Lack of energy was excluded owing to its high correlation with fatigue (r = 0.70). Among the remaining 8 symptoms, one-quarter of the correlation coefficients ranged from 0.31 (fatigue and bone pain) to 0.51 (pain and bone pain) while all other correlations were below r = 0.30. This interdependence should be considered when interpreting the mediation findings.

In addition, adjustments for multiple comparisons were not applied. The sample size available with PRO data was reduced at each time point compared with baseline. Disease progression was the main reason for discontinuation and, therefore, subsequent unavailability of PRO data.^12^^,^^13^ In particular, the sunitinib group had higher rates of discontinuation due to disease progression and toxicity.^12^^,^^13^ Therefore, the pattern of missing data may not be random, which could bias the mediation analysis estimates. For example, patients remaining in the trial and reporting outcomes may be more likely to maintain HRQOL than those who dropped out. As a consequence, the identified HRQOL improvements and the corresponding underlying contributing factors may be subject to survival benefits for staying on treatment longer. As such, this exploratory analysis was limited to week 49, when approximately one year of PRO data were available and sample sizes remained reasonable in both treatment arms. However, it was previously reported that HRQOL findings were not strongly dependent on the nature of the missing data.^13^ Lastly, as noted previously, the CheckMate 9ER trial was designed with different PRO data collection schedules across treatment arms: every 2 weeks in the nivolumab plus cabozantinib arm and every 6 weeks in the sunitinib arm. A higher frequency of data collection may prompt differences in the reporting of symptoms; therefore, to avoid potential biases, the current analysis was restricted to PRO data collected at the common 6-week intervals shared by both treatment arms.

## Conclusions

Clinical trials typically focus on the efficacy of novel treatments by studying direct effects on clinical outcomes. This analysis identified the underlying factors contributing to improvements in HRQOL with nivolumab plus cabozantinib, compared with sunitinib, in patients with aRCC in the CheckMate 9ER trial. Patients treated with nivolumab plus cabozantinib had increased HRQOL benefits compared with those receiving sunitinib, which was largely attributed to reduction in symptoms of fatigue, pain, cough, and shortness of breath, as well as improvements in emotional well-being. These findings shed light on the interpretation of PRO data from a clinical trial to inform how changes in the symptom manifestations of a disease can directly contribute to improved patient experiences captured by established PRO instruments. Such interpretation may provide further support for the use of standard-of-care nivolumab plus cabozantinib in patients with aRCC.

## Supplementary Material

oyag329_Supplementary_Data

## Data Availability

Individual participant data will not be made available.
