# Supplementary material for: Identification of key symptoms impacting HRQOL in advanced renal cell carcinoma: nivolumab plus cabozantinib versus sunitinib in CheckMate 9ER
Source: Oncologist. 2026 Aug 14;31(9):oyag329. doi: 10.1093/oncolo/oyag329 (PMC13537816; doi:10.1093/oncolo/oyag329)
Supplement: Supplementary file 1 [file oyag329_supplementary_data.zip › Bergerot_9ER PRO_Supplement.docx]

**Identification of key symptoms impacting HRQOL in advanced renal cell carcinoma: nivolumab plus cabozantinib versus sunitinib in CheckMate 9ER**

**SUPPLEMENTAL APPENDIX**

**Table S1.** **Correlation among FKSI-DRS symptoms at baseline**

|  | **Lack of energy** | **Pain** | **Weight loss** | **Fatigue** | **Shortness of breath** | **Fevers** | **Bone pain** | **Cough** | **Blood in urine** |
| --- | --- | --- | --- | --- | --- | --- | --- | --- | --- |
| **Lack of energy** | - | - | - | - | - | - | - | - | - |
| **Pain** | 0.47 | 1 | - | - | - |  | - | - | - |
| **Weight loss** | 0.39 | 0.38 | 1 | - | - | - | - | - | - |
| **Fatigue** | 0.70 | 0.47 | 0.38 | 1 | - | - | - | - | - |
| **Shortness of breath** | 0.45 | 0.23 | 0.24 | 0.48 | 1 | - | - | - | - |
| **Fevers** | 0.26 | 0.17 | 0.22 | 0.24 | 0.27 | 1 | - | - | - |
| **Bone pain** | 0.32 | 0.51 | 0.18 | 0.31 | 0.19 | 0.13 | 1 | - | - |
| **Cough** | 0.28 | 0.13 | 0.21 | 0.28 | 0.34 | 0.21 | 0.05 | 1 | - |
| **Blood in urine** | 0.08 | 0.03 | 0.14 | 0.06 | 0.04 | 0.07 | 0.08 | 0.10 | 1 |
